# When spectators feel ashamed: vicarious shame and behavioral responses to in-group misconduct

**DOI:** 10.3389/fpsyg.2026.1834709

**Published:** 2026-08-28

**Authors:** Lei Ren, Hong Ma, Xiaoli Zong

**Affiliations:** 1 Department of Physical Education, Shanxi Normal University, Taiyuan, China; 2 Department of Education, Taiyuan Normal University, Jinzhong, China; 3 Department of Medical Psychology, Army Medical University, Chongqing, China

**Keywords:** behavioral intentions, group identification, negative evaluation, sports spectators, vicarious shame

## Abstract

**Introduction:**

In the context of sport spectatorship, spectators may experience feelings of vicarious shame when witnessing their in-group members' misconduct, which can significantly impact their behavioral responses. Despite growing interest in this phenomenon, it remains unclear which factors impact the emergence of vicarious shame among spectators and how this emotion shapes subsequent behavioral intentions. The current study adopted a cross-sectional design to examine how sports spectators' negative evaluations of deviant in-group members were associated with their experience of vicarious shame, and in turn, their subsequent behavioral intentions (i.e., distance, repair, and punishment), as well as the moderating role of group identification in this process.

**Methods:**

Participants were 376 Chinese adult spectators who reported having experienced vicarious shame during sports competitions. The spectators self-reported on their negative evaluations, behavioral intentions, vicarious shame, and group identification. A moderated mediation model was conducted using structural equation modeling to test the hypothesized associations.

**Results:**

Results showed that vicarious shame mediated the positive associations between negative evaluation and all three behavioral intentions. Furthermore, group identification strengthened the positive association between negative evaluation and vicarious shame.

**Conclusion:**

These findings highlight vicarious shame as a multifaceted emotion that can serve important social and moral regulatory functions in group processes, providing valuable implications for moral education initiatives among sports spectators.

## 1 Introduction

Sports spectatorship is a highly social and identity-laden context. During sport events, spectators do not merely observe athletic performance; they often experience victories, defeats, and conflicts as members of a collective. In such settings, the boundary between the individual self and the group can become blurred, and the behavior of other in-group members can have implications for one's own social image. This is particularly relevant when spectators witness uncivil or misconduct behaviors by players of the team they support or other spectators supporting the same team, such as verbally abusing referees or opponents, insulting rival supporters, engaging in physical conflict, throwing objects onto the field, disrupting venue order, or displaying other norm-violating behaviors. Such incidents may not only disrupt the sporting environment but also elicit self-conscious, group-based emotions among observers.

One important emotion in this context is *vicarious shame*, which refers to the shame people experience in response to the wrongdoing or norm violation of others with whom they feel connected (Lickel et al., 2005; Tangney et al., 2007). In sport spectatorship, vicarious shame may be especially consequential because in-group misconduct often occurs in public, emotionally charged, and intergroup settings. Spectators who feel ashamed on behalf of their group may try to protect their self-image by distancing themselves from the situation, repairing the group's image, or supporting sanctions against the offending members (Partridge et al., 2020). Thus, vicarious shame is not merely a private emotional reaction, but a potentially important psychological process through which spectators respond to threats to group image and moral norms.

Despite growing interest in vicarious shame, important questions remain unexplored in the sport context. Prior research has shown that the perceived negativity of shame-eliciting *events* is associated with vicarious shame and coping responses (Johns et al., 2005). However, shame is closely tied to threats to self- and group image, suggesting that spectators' negative evaluations of the transgressing in-group members themselves may be particularly relevant. Moreover, because group-based emotions depend on the extent to which individuals define themselves through group membership, group identification may shape whether negative evaluations of in-group misconduct translate into vicarious shame. Clarifying these processes is important for understanding how spectators regulate group boundaries and moral norms, as well as how shame-related responses may contribute to both constructive and problematic forms of spectator behavior. The present study examined how spectators' negative evaluations of deviant in-group members were associated with vicarious shame and subsequent behavioral intentions, and whether this process was moderated by group identification.

### 1.1 Theoretical framework

Social identity theory and self-categorization theory provide the foundational framework for understanding why individuals may experience emotions in response to the actions of other group members. According to social identity theory, individuals define themselves not only in terms of personal attributes, but also through their membership in social groups, and such group memberships can become part of their self-concept (Tajfel and Turner, 1986). Because individuals are motivated to maintain a positive social identity, the image and status of the in-group can become closely tied to self-evaluation and self-esteem. Self-categorization theory further specifies the cognitive process through which a particular group identity becomes salient (Turner et al., 1987). When individuals categorize themselves as members of a group, they tend to perceive themselves and other in-group members as sharing a common identity. As a result, the actions of in-group members may acquire personal relevance because they are interpreted as reflecting, affirming, or threatening the group identity that forms part of the individual's self-concept.

These identity-based processes are particularly relevant in sport spectatorship. Sport events often make group boundaries highly salient, as spectators categorize themselves and others in terms of team affiliation, shared support, and opposition to rival groups. In such contexts, players and the spectators who support the team may be perceived as in-group members, and their conduct can have implications for the spectator's own social identity. Therefore, misconduct by in-group members may not be experienced merely as an external event, but as a threat to the image of the group with which the spectator identifies.

Building on social identity theory and self-categorization theory, intergroup emotions theory provides a more specific account of how group-based self-definition gives rise to emotions experienced on behalf of the group (Mackie et al., 2000; Mackie and Smith, 2018). According to this theory, when a social identity is salient, individuals appraise events in terms of their implications for the in-group, and these group-based appraisals elicit corresponding emotional and behavioral tendencies (Mackie and Smith, 2017). From this perspective, vicarious shame may arise when an in-group member's misconduct is appraised as damaging to the group's moral or social image. Spectators who witness uncivil behavior by in-group members may therefore feel ashamed because such behavior reflects negatively on a group that forms part of their self-concept.

### 1.2 Negative evaluation and vicarious shame

Based on the theoretical framework outlined above, spectators' negative evaluations may serve as a proximal appraisal through which in-group misconduct becomes emotionally consequential. Previous studies have examined the relationship between negative evaluations and vicarious shame. For instance, Daniels and Robinson's (2019) review in the organizational context showed that vicarious shame arises when a behavior is appraised as a negative deviation, discrepancy, or shortcoming from standards tied to one's work-related identity. They further identified two common types of behaviors that trigger vicarious shame: moral transgressions and violations of social norms. In the sport context, although a limited number of studies have explored the association between vicarious shame and subsequent coping experiences (Partridge and Wann, 2015; Partridge et al., 2020), little attention has been paid to the cognitive processes underlying vicarious shame. Partridge et al. (2020) found that the perceived negativity of events associated with vicarious shame was positively correlated with the motivation to distance oneself from the group. However, the appraisal of perceived negativity in this study focused on the evaluation of events rather than on the individuals who committed the transgressions. Given that shame is typically linked to threats to the image of an individual or one's group (Lewis, 1971), evaluations directed toward the offender may be more critical in triggering vicarious shame. Moreover, focusing on the evaluation of individuals provides a more direct account of how reputational concerns are internalized, as the offender serves as a representative of the group and embodies the threat to the group's image. This issue has remained largely unexamined in the sports domain, making it a primary focus of the present study.

### 1.3 Behavioral responses to vicarious shame

Vicarious shame can elicit subsequent behavioral responses. Existing research shows that distance and repair are the two most frequently investigated behavioral intentions in the context of vicarious shame. Distance refers to the desire to dissociate oneself from shameful events or group members in order to protect one's own image from negative evaluations. The positive association between distancing motivation and vicarious shame has been consistently supported (Figueroa-Caballero and Mastro, 2019; Johns et al., 2005; Partridge et al., 2020). In contrast, repair refers to the desire to restore the group's image through constructive efforts. Although this tendency has been shown to be more strongly related to vicarious guilt (Martinovic et al., 2021), scholars have argued that group-based shame can also stimulate a desire to change the group's image in a positive way (Lickel et al., 2007). In support of this view, empirical evidence from studies on colonization and racial discrimination has demonstrated that vicarious shame is positively associated with the attitude toward compensating harmed groups (Allpress et al., 2010; Brown and Cehajic, 2008; Gausel et al., 2012), suggesting that vicarious shame may contain a more prosocial and constructive aspect compared to personal shame (Tangney et al., 2007).

In addition to distance and repair, another important yet underexplored behavioral intention that may be closely related to vicarious shame is punishment, which refers to the desire to sanction or harm in-group members for eliciting vicarious shame. Punishment is typically conceptualized as an affective response related to anger (Iyer et al., 2007) and has received little attention in vicarious shame research. The relevance can be reflected through the black sheep effect, which indicates that individuals judge in-group members who damage the group's reputation more harshly than out-group members (Marques et al., 1988). Supporting this view, Chekroun and Nugier (2011) found that when an in-group's social image was threatened by members' deviant behavior, other group members experienced heightened vicarious shame and were more likely to engage in punitive behaviors toward the deviant.

In the sports context, research on behavioral intentions associated with vicarious shame remains scarce. Existing studies have typically examined these intentions in isolation, without systematically addressing their distinct effects (Costabile and Austin, 2018; Partridge et al., 2020). More importantly, it is unclear whether spectators' experiences of vicarious shame primarily foster prosocial in-group behavioral constraints, such as regulating group members' behavior and preserving the group's image, or whether they may also escalate into large-scale negative collective behaviors, such as riots. This gap underscores the need for further empirical investigation into the diverse behavioral consequences of vicarious shame in sports settings.

### 1.4 The moderating role of group identification

Although the negative evaluation of in-group members who conducted transgressions is a critical antecedent of vicarious shame, not all individuals who negatively evaluate in-group members necessarily experience the same level of vicarious shame. The intergroup emotion theory indicates that the association between negative evaluation and vicarious shame may depend on the extent to which individuals identify with the group. Specifically, when individuals strongly identify with a group, their self-concept is closely tied to the group membership, as a result, they are more likely to experience emotions on behalf of the group (Smith and Henry, 1996).

Empirical evidence supports this moderating role of group identification in the association between negative evaluation and vicarious shame. For example, Johns et al. (2005) examined American college students' experiences of vicaious shame for other Americans who engaged in anti-Arab prejudice after the 9/11 attacks. They found that the event negativity and identification with being American had an interactive effect on vicarious shame. However, their study focused on event negativity as the moderator rather than directly testing the moderating effect of group identification, leaving open the question of whether and how group identification may shape the link between negative evaluations of in-group members and vicarious shame.

### 1.5 The present study

**Figure 1:**
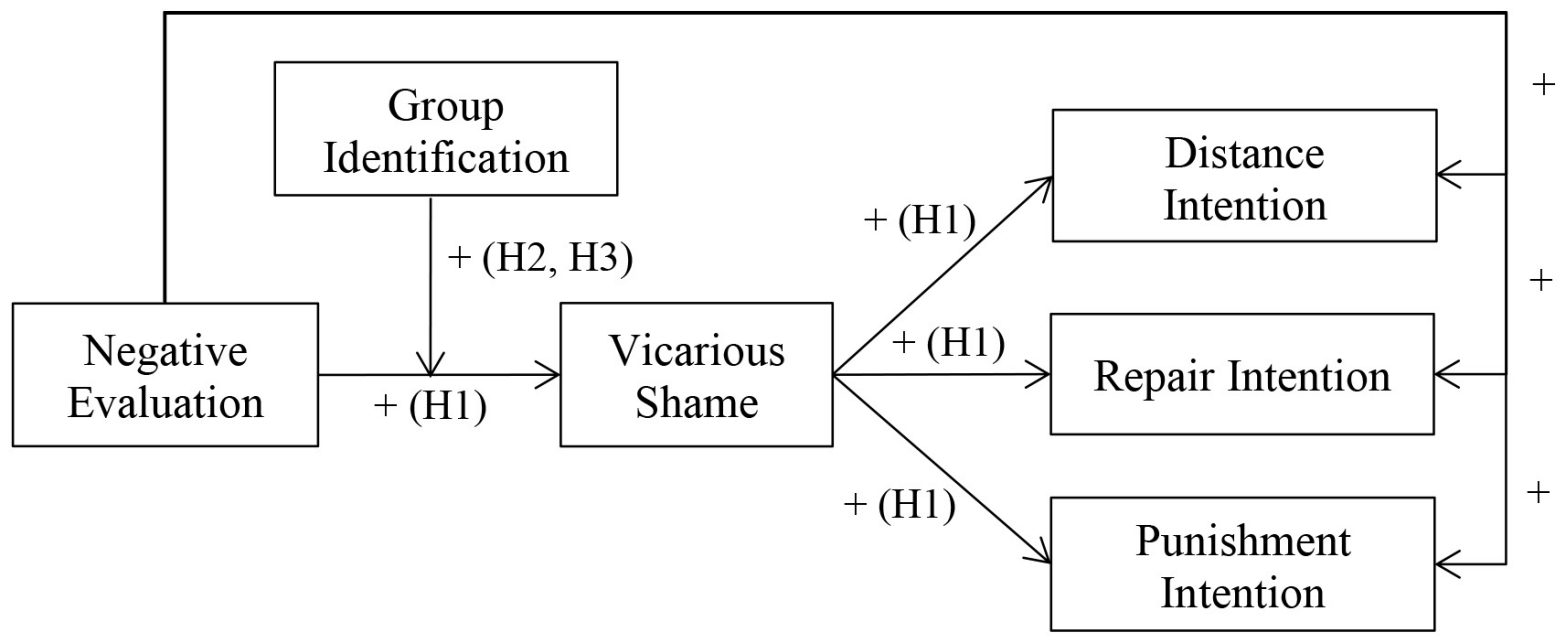
The proposed moderated mediation model. Plus signs (+) indicate hypothesized positive associations. H1 = hypothesis 1, H2 = hypothesis 2, H3 = hypothesis 3.

The first aim of the present study was to examine whether sports spectators' negative evaluations of in-group members who conducted misbehaviors were associated with their vicarious shame and subsequent behavioral intentions (i.e., distance, repair, and punishment). The second aim was to explore the moderating role of group identification in the association between negative evaluations and vicarious shame. Figure 1 presents the conceptual diagram of the hypothesized model. Based on previous research (Chekroun and Nugier, 2011; Gausel et al., 2012; Partridge et al., 2020), we proposed the following hypotheses:

**Hypothesis 1:** Vicarious shame mediates the link between negative evaluations and the three behavioral intentions (i.e., distance, repair, and punishment) in sports spectators. Specifically, more negative evaluations of in-group members are associated with higher levels of vicarious shame, which in turn, are associated with stronger intentions of distance, repair, and punishment.

**Hypothesis 2:** Group identification moderates the association between negative evaluations and vicarious shame. Specifically, the positive association between negative evaluations and vicarious shame is stronger among spectators with higher levels of group identification.

**Hypothesis 3:** The indirect associations between negative evaluations and the three behavioral intentions through vicarious shame are conditional on group identification. Specifically, these indirect associations are stronger among spectators with higher levels of group identification.

## 2 Method

### 2.1 Participants and procedure

A total of 376 Chinese adults participated in the study. All participants reported having experienced vicarious shame elicited by other in-group members' misconducts while watching sports competitions. The majority of participants were aged between 19 and 25 years (67.3%, *n* = 253), and 55.1% (*n* = 207) resided in Shanxi Province, an inland region in northern China. Most participants had a bachelor's degree (62.2%, *n* = 234), and 64.4% (*n* = 242) were students. More than half of the participants (57.2%, *n* = 215) reported attending live games fewer than three times a year. Detailed demographic characteristics are presented in Table 1.

**Table 1:** Demographic characteristics (*N* = 376).

Variable	*n*	*%*
Sex		
Female	158	42.0
Male	218	58.0
Age^a^		
19–25 years	253	67.3
26–35 years	97	25.8
36–45 years	16	4.3
>45 years	10	2.7
Province of residence^b^		
Shanxi	207	55.1
Other	169	45.9
Levels of education		
High school or below	12	3.2
Bachelor's degree	234	62.2
Master's degree or above	130	34.6
Occupation		
Student	242	64.4
Employee of a company or institution	87	23.1
Self-employed/Freelance worker	31	8.2
Other	16	4.3
Frequency of attending live games per year		
< 3 times	215	57.2
3–5 times	70	18.6
6–10 times	42	11.2
11–20 times	23	6.1
21–30 times	6	1.6
>30 times	20	5.3

^a^Age was assessed using four predefined categories: 19–25 years, 26–35 years, 36–45 years, and >45 years.

^b^Province of residence was based on participants' self-reported residential location; because responses outside Shanxi Province were sparse, all non-Shanxi responses, including four overseas responses, were combined into an “Other” category.

The participants were recruited through flyers distributed via social media platforms (e.g., WeChat). Potential participants were informed of the study objectives and procedures and provided online informed consent. They were then screened for eligibility. First, they were asked whether they had attended any formal sports events in person during the past year, excluding events watched only through television or online media. Those who answered yes were further asked whether they had ever felt ashamed because of uncivil or misconduct behavior by players or spectators of the teams they supported during such events. Only participants who reported both in-person spectating experience and shame-related experience were invited to continue the survey.

The study used a retrospective event-recall approach rather than a vignette-based design. Participants were instructed to recall a real scene from an in-person sports event in which players or spectators of the teams they supported engaged in uncivil or shameful misconduct, and to answer the subsequent questions based on their thoughts and feelings at that time. To avoid priming participants with predefined categories of misconduct, we did not provide specific behaviors or examples, so that participants could draw on naturally occurring experiences that they themselves perceived as shame-eliciting. Thus, vicarious shame was operationalized as participants' self-reported shame in response to recalled misconduct by in-group members in an actual sport spectating context.

The survey was anonymous and took approximately 12 min to complete. Upon completion, participants were entered into a lucky draw for cash prize.

### 2.2 Measures

#### 2.2.1 Negative evaluation

Participants' negative evaluations of the in-group members who engaged in misbehaviors were assessed using four items adopted from a previous study with Chinese participants (Ma and Ren, 2023). Based on their recalled experiences of vicarious shame during sport spectatorship, participants rated on four items (“I had a negative impression of this person / these people” “I thought I was more civilized than this person / these people” “I thought this person / these people should not have been part of my group” “I thought this person / these people were uncivilized”). The items were rated on a 5-point Likert scale (1 = strongly disagree to 5 = strongly agree). The average scores were calculated, with higher scores representing more negative evaluation of the spectators who exhibit uncivil behaviors. The Cronbach's α was 0.80 in the present study.

#### 2.2.2 Group identification

Participants' group identification as sports spectators was measured using the 6-item Organizational Identification Scale (Mael and Ashforth, 1992), which has been validated in Chinese culture (Uen et al., 2011). To adapt the measure to the sport spectatorship context, participants were instructed to answer the items with reference to the group comprising themselves, the team they supported, or other spectators supporting the same team. The items include, “When someone criticizes my group, it feels like a personal insult.” “I am very interested in what others think about my group.” “When I talk about my group, I usually say ‘we' rather than ‘they'.” “My group's successes are my successes.” “When someone praises my group, it feels like a personal compliment.” “If a story in the media criticized my group, I would feel embarrassed.” All items were rated on a 5-point Likert scale (1 = strongly disagree to 5 = strongly agree). The average scores were calculated, with higher scores representing stronger group identification. The Cronbach's α was 0.82 in the present study.

#### 2.2.3 Vicarious shame

Participants' vicarious shame in response to the uncivil behaviors of other in-group members was measured using five items from the Vicarious Shame Scale for Sports Spectators, which developed and validated in a previous study with Chinese participants (Ma and Ren, 2023). The items included “I felt ashamed” “I felt awkward” “I felt embarrassed” “I felt humiliated” “I felt that I had lost face.” All items were rated on a 5-point Likert scale (1 = strongly disagree to 5 = strongly agree). The average scores were calculated, with higher scores representing more intense emotional experience. The Cronbach's α was 0.88 in the present study.

#### 2.2.4 Behavioral intentions

Participants' behavioral intentions in response to feeling ashamed due to in-group misconduct were assessed using items adopted from a previous study with Chinese participants (Ma and Ren, 2023). Three types of intended responses were assessed: distancing, repair, and punishment. Distance was measured using three items, “I wanted to get away from the scene quickly” “I wanted to avoid eye contact with others” “I did not want to acknowledge that this person / these people were responsible for such behavior.” Repair was measured using three items, “I wanted to explain to this person / these people that their behavior was inappropriate” “I wanted to stop this person / these people from continuing such behavior” “I wanted to apologize to those who were harmed by this person's / these people's behavior.” Punishment was measured using two items (“I want to drive this person / these people away” “I wanted to call the police or have security remove this person / these people”). All items were rated on a 5-point Likert scale (1 = strongly disagree to 5 = strongly agree). The average scores were calculated for each behavioral intention, with higher scores representing stronger intentions. The Cronbach's αs of the distance, repair, and punishment intentions were 0.71, 0.77 and 0.78 in the present study, respectively.

### 2.3 Data analysis

A moderated mediation model was conducted using structural equation modeling (SEM) to examine the mediating role of vicarious shame in the association between negative evaluation and behavioral intentions of distance, repair, and punishment, as well as the moderating role of group identification in the association between negative evaluation and vicarious shame. Participants' sex and age were included as covariates in the analyses because they showed significant associations with the study variables (see Table 2). Sex was dummy-coded (0 = male, 1 = female). Age was treated as an ordinal approximation in the statistical model (1 = 19–25 years, 2 = 26–35 years, 3 = 36–45 years, 4 = >45 years).

**Table 2:** Means, standard deviations, and bivariate correlations of study variables.

Variable	1	2	3	4	5	6	7	8
1. Negative evaluation	–							
2. Group identification	0.19^***^	–						
3. Vicarious shame	0.48^***^	0.30^***^	–					
4. Distance intention	0.43^***^	0.27^***^	0.69^***^	–				
5. Repair intention	0.41^***^	0.26^***^	0.53^***^	0.54^***^	–			
6. Punishment intention	0.67^***^	0.22^***^	0.47^***^	0.47^***^	0.49^***^	–		
7. Sex^a^	−0.14^**^	0.10^*^	0.01	0.00	0.08	−0.11^*^	–	
8. Age^b^	0.23^***^	−0.07	0.01	−0.02	−0.13^*^	0.09	−0.06	–
*M*	3.44	3.62	3.13	3.33	2.90	2.78	–	–
*SD*	0.85	0.75	0.90	0.86	0.91	1.04	–	–

^a^Male = 0, Female = 1.

^b^19–25 years old = 1, 26–35 years old = 2, 36–45 years old = 3, over 45 years old = 4.

^*^p < 0.05.

^**^p < 0.01.

^***^p < 0.001.

Given that the distributions of study variables in our sample were approximately normal (kurtosis ranged from −0.54 to 0.66, skewness ranged from −0.61 to 0.23), the maximum likelihood (ML) estimator was used to estimate the model. A bootstrapping approach with 5,000 random samples was used to test the indirect effects. When there was a significant moderating effect, simple slope analyses were conducted at low (−1 SD), mean, and high (+1 SD) levels of group identification to probe the pattern of the moderation. We also examined the conditional indirect effects of negative evaluation on behavioral intentions through vicarious shame across different levels of group identification (Hayes, 2009).

Model fit was evaluated using the chi-square statistic (χ^2^), the comparative fit index (CFI), the root mean square error of approximation (RMSEA), and the standardized root mean square residual (SRMR). Good model fit was indicated by CFI > 0.95, RMSEA < 0.06, and SRMR < 0.08, and acceptable model fit was indicated by CFI > 0.90, RMSEA < 0.08, and SRMR < 0.10 (Hu and Bentler, 1999). The SEM was conducted using Mplus Version 8.3. The missing rate of the data was less than 1%. Missing data were handled using Full Information Maximum Likelihood (FIML), which utilizes all available data to provide unbiased estimates (Enders and Bandalos, 2001).

To ensure adequate statistical power for evaluating the overall fit of the SEM, we first conducted an a priori power analysis using the semPower package (Moshagen and Erdfelder, 2016) in R. Assuming α = 0.05 and power = 0.80, the analysis indicated that a minimum sample size of 356 participants was required to achieve acceptable model fit (RMSEA ≤ 0.08). In addition to overall model fit, we further examined the minimum sample size required to detect the hypothesized moderation effect, which represents a key structural path and is typically among the smallest effect sizes in SEM. Specifically, we conducted a separate a priori power analysis for the interaction term using the InteractionPoweR package (Baranger et al., 2023) in R. Parameter estimates were informed by prior research (Johns et al., 2005), with correlations of *r* = 0.44 between negative evaluation and vicarious shame, *r* = 0.00 between negative evaluation and group identification, and *r* = 0.29 for their interaction predicting vicarious shame. Given the nonsignificant main effect of group identification reported previously, we conservatively specified this correlation as *r* = 0.10. With α = 0.05 and power = 0.80, the required sample size for detecting the interaction effect was estimated to be 80 participants. Together, these analyses indicate that the planned sample size was sufficient to ensure both acceptable model fit and adequate power for detecting the focal interaction effect.

## 3 Results

### 3.1 Descriptive statistics and correlation analysis

Means, standard deviations, and the correlations among study variables are shown in Table 2. Negative evaluation, vicarious shame and distance, repair, and punishment behavioral intentions were all significantly and positively correlated with one another. Females reported higher levels of group identification and lower levels of negative evaluation and punishment intentions compared to males. Age was positively correlated with negative evaluation and negatively correlated with distance intention.

### 3.2 The moderated mediation model

**Figure 2:**
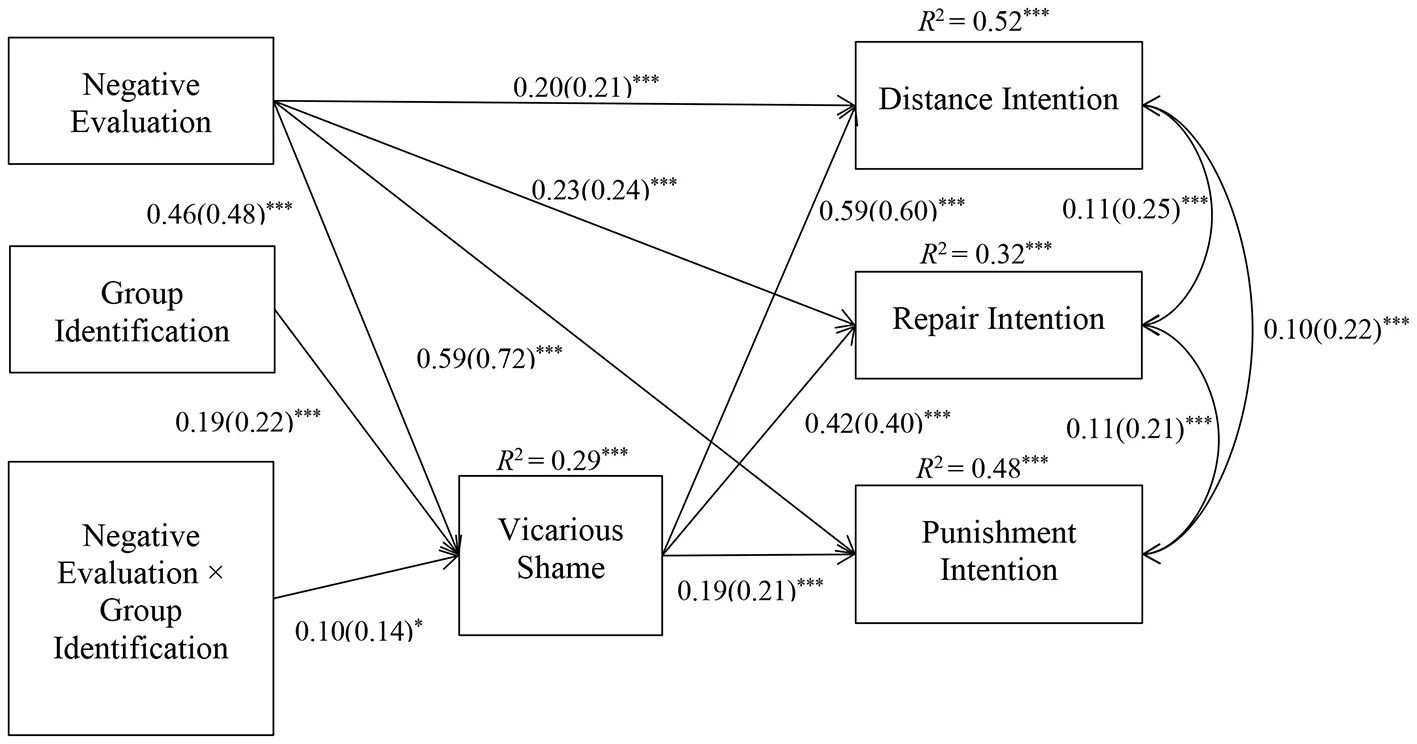
Group identification moderated the indirect associations between negative evaluation and behavioral intentions through vicarious shame. Standardized regression coefficients are presented. Unstandardized regression coefficients are presented in parentheses. Covariates are sex and age. *p < 0.05, ***p < 0.001.

The moderated mediation model achieved a good fit: χ^2^ (6, *N* = 376) = 6.64, *p* = 0.356, CFI = 0.99, RMSEA = 0.02, 90% CI [0.00, 0.07], SRMR = 0.02. The standardized path coefficients are shown in Figure 2. After controlling for participants' sex and age, the direct effects of negative evaluation on distance intention (*β* = 0.20, *p* < 0.001), repair intention (*β* = 0.23, *p* < 0.001), and punishment intention (*β* = 0.59, *p* < 0.001) were all significant and positive. As expected, negative evaluation was positively associated with vicarious shame (*β* = 0.46, *p* < 0.001), which in turn, was positively associated with the three behavioral intentions (*β*_distance_ = 0.59, *p* < 0.001; *β*_repair_ = 0.42, *p* < 0.001; *β*_punishment_ = 0.19, *p* < 0.001). To better understand these associations, we compared the strength of the link between vicarious shame and the three behavioral intentions, as well as the the link between negative evaluation and the three behavioral intentions using Wald tests. The link between vicarious shame and distance was significantly stronger than the links with repair (*p* = 0.001) and punishment (*p* < 0.001), and the association between vicarious shame and repair was stronger than that with punishment (*p* = 0.006). The link between negative evaluation and punishment was significantly stronger than the links with distance and repair (*ps* < 0.001), but the associations between negative evaluation and distancing vs. repair did not differ (*p* = 0.74).

**Figure 3:**
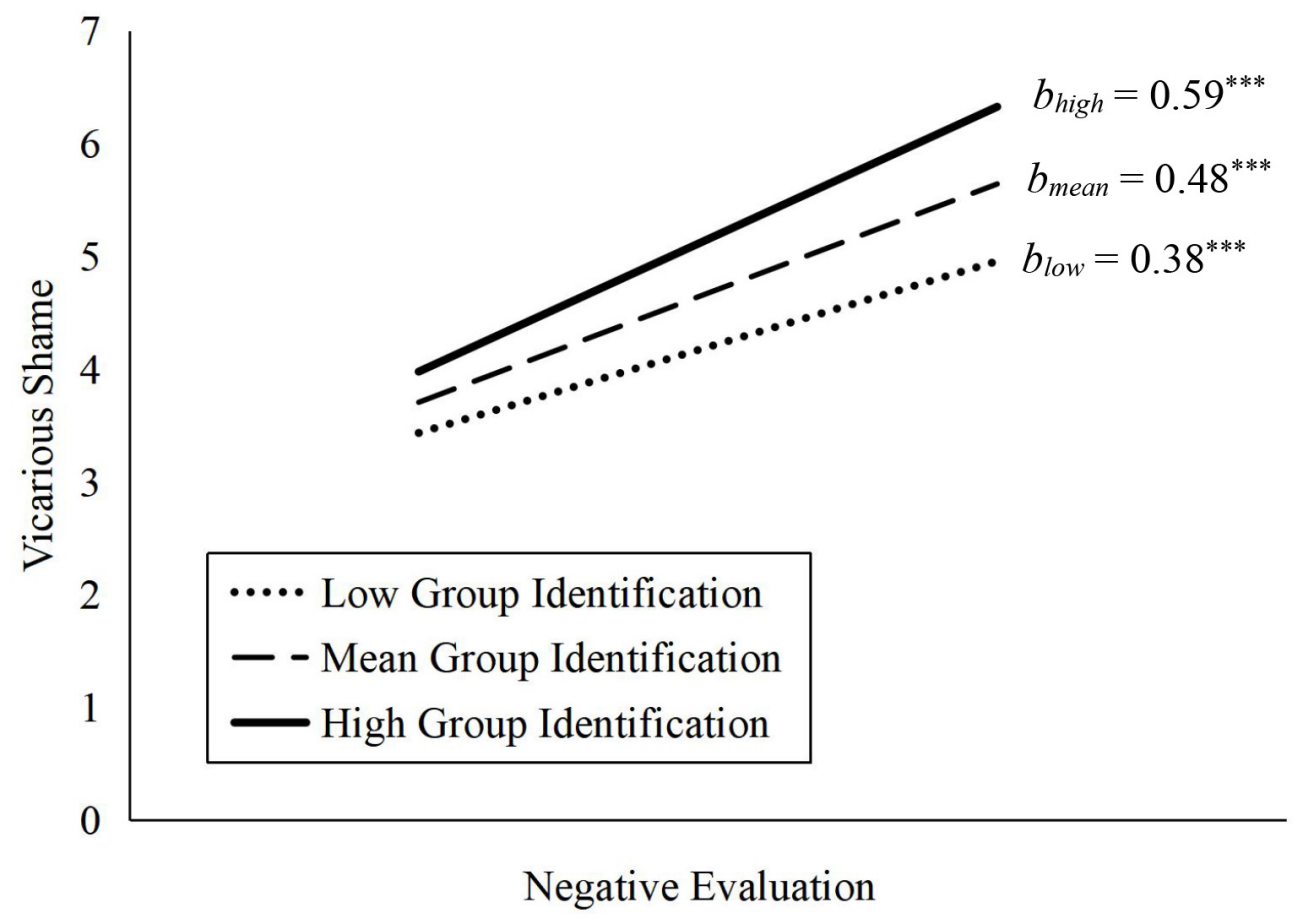
The associations between negative evaluation and vicarious shame at low (−1 SD), mean, and high (+1 SD) levels of group identification. ***p < 0.001.

The link between negative evaluation and vicarious shame was further moderated by group identification (*β* = 0.14, *p* = 0.038). As shown in Figure 3, although this association was significant across all three levels of group identification, the strength of the association increased with higher levels of group identification. In addition, the indirect effects of negative evaluation on three behavioral intentions through vicarious shame were all significant and positive across low, mean, and high levels of group identification (Table 3), with these indirect effects becoming stronger as group identification increased.

**Table 3:** Indirect effects of negative evaluation on behavioral intentions at different levels of group identification.

Levels of group identification	Indirect effect (*SE*)		
	Distance intention	Repair intention	Punishment intention
Low (−1 *SD*)	0.23 (0.05)	0.15 (0.04)	0.08 (0.03)
	95% CI [0.13, 0.33]	95%CI [0.09, 0.23]	95%CI [0.04, 0.15]
Mean	0.29 (0.04)	0.19 (0.03)	0.10 (0.03)
	95%CI [0.22, 0.36]	95%CI [0.13, 0.26]	95%CI [0.05, 0.17]
High (+1 *SD*)	0.35 (0.04)	0.23 (0.04)	0.13 (0.03)
	95%CI [0.27, 0.44]	95%CI [0.16, 0.32]	95%CI [0.07, 0.20]

SD, standard deviation; SE, standard error; CI, confidence interval.

## 4 Discussion

The present study investigated the associations among sports spectators' negative evaluations of in-group members who engaged in misbehaviors during sport events, their experiences of vicarious shame, and three subsequent behavioral intentions (i.e., distance, repair, and punishment). We also examined the moderating role of group identification in this process. We found that spectators who evaluated deviant in-group members more negatively experienced higher levels of vicarious shame, which in turn, were associated with stronger intentions to distance themselves from the situation, to restore the group's image, and to punish the individuals who did misbehaviors within the group. Furthermore, group identification strengthened the positive association between negative evaluation and vicarious shame.

### 4.1 Mediating role of vicarious shame

Consistent with Hypothesis 1, vicarious shame partially mediated the associations between negative evaluations and all three behavioral intentions. Specifically, spectators who evaluated deviant in-group members more negatively reported stronger vicarious shame, which in turn was associated with stronger intentions to distance themselves, repair the group's image, and punish the offending members. At the same time, the direct effects of negative evaluation on the three behavioral intentions remained significant, indicating that vicarious shame did not fully account for these associations and that other mechanisms may also be involved.

These findings can be understood within the broader social identity approach. Social identity theory (Tajfel and Turner, 1986) and self-categorization theory (Turner et al., 1987) suggest that when a group identity becomes salient, individuals define themselves partly through group membership and evaluate events in terms of their implications for the in-group. In sport spectatorship, the public and emotionally charged nature of competition may make the identity of being a supporter especially salient. Under such conditions, the misconduct of same-team supporters or other in-group members can be interpreted as a threat to the moral and social image of a group that forms part of the spectator's self-concept. This helps explain why negative evaluations of deviant in-group members were associated with stronger vicarious shame. In addition, our findings also extend intergroup emotions theory (Mackie et al., 2000) by showing that vicarious shame may function as an identity-threatening group-based emotion in sport spectatorship, which arises when spectators perceive in-group misconduct as damaging to the group's image and is subsequently linked to different behavioral intentions (Mackie et al., 2000).

Among the three behavioral intentions, vicarious shame was most strongly associated with distance. From a self-categorization perspective (Turner et al., 1987), distancing may serve as a way to reduce identity threat by symbolically separating the self from the deviant member or the shame-eliciting event. This finding is consistent with the well-documented phenomenon of “cutting off reflected failure” in the sport fan literature, which describes how individuals protect their self-image by dissociating from failed or deviant in-group members (Larkin et al., 2022; Wann et al., 1995).

Vicarious shame was also associated with repair intention. Although shame has often been linked to maladaptive outcomes (Chen et al., 2025; Kapadia et al., 2025), our findings suggest that shame in group contexts may also motivate efforts to restore a damaged group image. From a social identity perspective, reparative responses can be understood as attempts to protect or restore a positive social identity after in-group misconduct. Supporting this view, prior work showed that when group members perceive an opportunity to improve the group's image, reparative strategies can alleviate vicarious shame and help restore the group's image (Brown et al., 2008; Brown and Cehajic, 2008).

Vicarious shame showed a relatively weak but significant association with punishment intention. From a social identity perspective, punitive responses may serve a boundary-regulating function. By sanctioning deviant in-group members, spectators may attempt to signal that the misconduct does not represent the group as a whole and to reaffirm the group's moral standards. This interpretation is consistent with the black sheep effect (Marques et al., 1988), which suggests that deviant in-group members may be judged harshly when they threaten the positive image of the group. Importantly, however, the direct effect of negative evaluation on punishment intention was the largest among the three behavioral intentions, whereas the indirect effect through vicarious shame was the smallest. This pattern suggests that punishment may be driven more directly by evaluative and normative processes, such as moral disapproval, perceived norm violation, anger, or moral outrage, rather than by shame itself (Hofmann et al., 2014). Therefore, vicarious shame appears to play a less central mediating role in punishment than in distancing and repair, highlighting the need to examine additional emotional and moral mechanisms in future research.

The findings of the present study suggest that identifying the conditions under which spectators are more likely to adopt distancing, reparative, or punitive responses following experiences of vicarious shame is an important direction for future research. Future studies could examine whether different responses are associated with distinct appraisals and situational cues, such as perceived self-image threat, perceived possibility of repairing the group's image, perceived responsibility, and severity of misconduct (Brown et al., 2008; Brown and Cehajic, 2008; Hofmann et al., 2014; Larkin et al., 2022; Wann et al., 1995). For example, spectators may be more likely to distance themselves when the misconduct strongly threatens their personal or group image, whereas reparative responses may be more likely when spectators believe that the group's image can still be restored or that they have some responsibility to respond constructively. Punitive responses, by contrast, may be more likely when misconduct is perceived as severe or as a clear violation of group norms. Examining these possibilities would help clarify the appraisal processes through which spectators adopt different responses to in-group misconduct.

### 4.2 Moderating role of group identification

Our study further revealed that group identification strengthened the indirect association between negative evaluation of the deviant in-group members and spectators' behavioral intentions through vicarious shame. From the perspective of social identity theory and self-categorization theory, group membership becomes psychologically consequential when individuals define themselves in terms of that group identity. For spectators with stronger group identification, misconduct by in-group members is more likely to be interpreted as self-relevant and as threatening to the group's moral image; in contrast, spectators with weaker group identification may still disapprove of the misconduct, but such behavior is less likely to threaten their own social identity.

Schmader and Lickel (2006) argued that vicarious shame is more likely to arise when the negative behavior of group members is perceived as closely tied to group identity, which may be especially salient in the competitive and emotionally charged context of sport spectatorship. Consequently, highly identified spectators may perceive deviant in-group members as representing the group, making their misconduct more damaging to the spectator's self-concept and more likely to elicit vicarious shame (Chi et al., 2015). Because group identification also motivates members to maintain a positive group image (Mael and Ashforth, 1992; Villicana et al., 2018), vicarious shame may further translate into stronger intentions to distance from the shame-eliciting event, repair the group's image, or punish the offending members. Taken together, these findings suggest that group identification amplifies the emotional and behavioral consequences of negative evaluations of deviant in-group members by increasing the self-relevance of group-image threats.

### 4.3 Limitations and future directions

Several limitations of the present study should be acknowledged. First, most participants in our sample were aged between 19 and 25 years and residing in an inland region of northern China, which may limit the generalizability of the findings to spectators from other age groups, regions, or cultural backgrounds. In particular, the present study was conducted in a Chinese cultural context, where interdependence, face concern, and group image may be relatively salient. These cultural features may make spectators more sensitive to misconduct by in-group members and more likely to experience vicarious shame in response to threats to group image (Stipek, 1998). Therefore, the associations among negative evaluation, vicarious shame, and behavioral intentions may differ in less interdependent cultural contexts. Future research should replicate the present findings in more diverse and cross-cultural samples and directly examine whether cultural orientation, interdependence, or face concern moderates these processes.

Second, as the present study was exploratory in nature, our primary aim was to investigate the general mechanisms underlying vicarious shame in the spectator context, and we therefore did not distinguish between different types of spectators. However, general sports spectators and fans may differ in meaningful ways (Tamir et al., 2024). For highly identified sport fans, whose supporter role constitutes a central aspect of their self-concept, the strategy of “cutting off reflected failure” may be less applicable (Rees et al., 2015). Therefore, future research should further distinguish between these subgroups, as their different cognitive and emotional connections with the group may lead to different emotional and behavioral consequences.

Third, as this study adopted a cross-sectional and retrospective event-recall design, the temporal and causal ordering among negative evaluation, vicarious shame, group identification, and behavioral intentions cannot be firmly established. In addition, vicarious shame was not experimentally induced or manipulated; participants recalled a past sport spectating situation and reported their feelings and intended responses based on that recalled event. Although this approach captured naturally occurring experiences of vicarious shame, retrospective self-reports may be affected by memory bias and may not fully reflect the emotional intensity and situational dynamics at the time of the event. Future research would benefit from experimental designs that manipulate key predictors, such as the severity of in-group misconduct or the salience of group identification, to more directly test their causal effects on vicarious shame and subsequent behavioral intentions. Longitudinal designs would also be valuable for clarifying how responses to vicarious shame unfold over time.

Finally, although the present study focused on vicarious shame as a key mechanism because of its close links to shared social identity and self-image threat (Lickel et al., 2005; Tangney and Dearing, 2002), the findings also suggest that other emotional, cognitive, or moral processes may be relevant. The significant direct effects of negative evaluation on all three behavioral intentions indicate that vicarious shame does not fully explain how negative evaluations are translated into spectators' responses to deviant in-group members. This issue may be particularly important for punishment intention, for which the relatively strong direct effect suggests the need to consider additional mechanisms, such as anger, moral condemnation, or perceived norm violation (Hofmann et al., 2014). In addition, the relatively lower variance explained in repair intention suggests that reparative responses may depend on additional prosocial mechanisms, such as sympathy toward affected out-group members, perceived responsibility, or vicarious guilt (Martinovic et al., 2021). Vicarious guilt may be especially important to examine, given that shame and guilt often co-occur and may not always be clearly differentiated by participants (Tangney, 1991; Tangney et al., 2007). Future studies should measure vicarious shame and vicarious guilt simultaneously to disentangle their unique and shared effects on spectators' behavioral intentions, while also examining other potential mechanisms that may more fully explain spectators' responses to deviant in-group members.

## 5 Conclusions and implications

This study contributes to the literature by examining vicarious shame as a key psychological mechanism that links sports spectators' negative evaluations toward misbehaving in-group members and their subsequent behavioral intentions. Extending prior work that has focused primarily on negative evaluations of shame-eliciting events (Johns et al., 2005; Shepherd et al., 2013), the present study highlights the importance of negative evaluations directed toward the deviants. Specifically, spectators' evaluations of deviant in-group members may serve as a proximal appraisal through which group-image threats are internalized and translated into vicarious shame. Furthermore, group identification amplified the association between negative evaluations and vicarious shame, suggesting that highly identified spectators may be especially vulnerable to experiencing vicarious shame when in-group members' misconduct threatens the moral and social image of the group.

Importantly, by simultaneously examining three theoretically distinct behavioral intentions (i.e., distance, repair, and punishment) within a single model, the present study shows that vicarious shame is differentially associated with spectators' responses to in-group misconduct. Vicarious shame showed the strongest association with distancing intention, suggesting particular relevance for self-protective responses aimed at reducing identity threat. Vicarious shame was also associated with repair intention, indicating that shame in group contexts may motivate constructive efforts to restore the group's damaged image. The weaker association with punishment intention suggests that punitive responses may depend more strongly on additional evaluative or moral processes beyond shame. Taken together, these findings indicate that vicarious shame is a multifaceted group-based emotion that may contribute to both self-protective and image-restorative responses in the context of in-group misconduct.

The findings have practical implications for spectator management and moral education in sport contexts. Sport spectatorship is a public, emotionally charged, and intergroup setting in which in-group misconduct can quickly become a threat to group image and crowd regulation. Therefore, interventions should not only aim to reduce uncivil behavior itself, but also guide other spectators' emotional and behavioral responses after in-group misconduct occurs. Sport organizations and event managers may help spectators recognize wrongdoing while channeling identity-threatening emotions into constructive forms of group regulation, such as image repair and norm reinforcement. For example, drawing on in-group resources such as group affirmation can help buffer threats to collective identity while reducing excessive or aggressive reactions (Villicana et al., 2018). In addition, incorporating moral education into spectator programs may enhance responsible sports viewing by encouraging spectators to remain calm, exercise self-control, and critically evaluate in-group misconduct without resorting to impulsive or extreme behaviors. Such strategies may help preserve the positive functions of strong group identification while mitigating its potential risks in the context of sport spectatorship.

## Data Availability

The raw data supporting the conclusions of this article will be made available by the authors, without undue reservation.
